# Variations to plasma H_2_O_2_ levels and TAC in chronical medicated and treatment-resistant male schizophrenia patients: Correlations with psychopathology

**DOI:** 10.1038/s41537-024-00468-y

**Published:** 2024-04-11

**Authors:** Haidong Yang, Wenxi Sun, Man Yang, Jin Li, Jing Zhang, Xiaobin Zhang

**Affiliations:** 1 Department of Psychiatry, The Fourth People’s Hospital of Lianyungang, The Affiliated KangDa College of Nanjing Medical University, Lianyungang, 222003 China; 2 Institute of Mental Health, Suzhou Psychiatric Hospital, The Affiliated Guangji Hospital of Soochow University, Suzhou, 215137 China

**Keywords:** Schizophrenia, Biomarkers

## Abstract

Accumulating evidence suggests that imbalanced oxidative stress (OS) may contribute to the mechanism of schizophrenia. The aim of the present study was to evaluate the associations of OS parameters with psychopathological symptoms in male chronically medicated schizophrenia (CMS) and treatment-resistant schizophrenia (TRS) patients. Levels of hydrogen peroxide (H_2_O_2_), hydroxyl radical (·OH), peroxidase (POD), α-tocopherol (α-toc), total antioxidant capacity (TAC), matrix metalloproteinase-9 (MMP-9), and tissue inhibitor of metalloproteinases-1 (TIMP-1) were assayed in males with CMS and TRS, and matched healthy controls. Schizophrenia symptoms were assessed using the Positive and Negative Syndrome Scale (PANSS). The results demonstrated significant differences in the variables H_2_O_2_ (*F* = 5.068, *p* = 0.008), ·OH (*F* = 31.856, *p* < 0.001), POD (*F* = 14.043, *p* < 0.001), α-toc (*F* = 3.711, *p* = 0.027), TAC (*F* = 24.098, *p* < 0.001), and MMP-9 (*F* = 3.219, *p* = 0.043) between TRS and CMS patients and healthy controls. For TRS patients, H_2_O_2_ levels were correlated to the PANSS positive subscale (*r* = 0.386, *p* = 0.032) and smoking (*r* = −0,412, *p* = 0.021), while TAC was significantly negatively correlated to the PANSS total score (*r* = −0.578, *p* = 0.001) and POD and TAC levels were positively correlated to body mass index (*r* = 0.412 and 0.357, *p* = 0.021 and 0.049, respectively). For patients with CMS, ·OH levels and TAC were positively correlated to the PANSS general subscale (*r* = 0.308, *p* = 0.031) and negatively correlated to the PANSS total score (*r* = −0.543, *p* < 0.001). Furthermore, H_2_O_2_, α-toc, and ·OH may be protective factors against TRS, and POD was a risk factor. Patients with CMS and TRS exhibit an imbalance in OS, thus warranting future investigations.

## Introduction

Schizophrenia is a chronic, severe, and often life-long mental disorder in which approximately half of patients do not respond well to medication, resulting in significant functional deficits^1,2^. Although atypical antipsychotics can be used to treat schizophrenia, 20–50% of patients are defined as having treatment-resistant schizophrenia (TRS), resulting in increased costs by 3−11 fold as compared to patients in remission^3,4^. Furthermore, clozapine is prescribed 8% more often for men with TRS than women, indicating the prevalence of TRS among males^5,6^. However, the underlying etiopathology of TRS remains relatively unknown, although interactions among multiple etiologies are suspected.

**Fig. 1:**
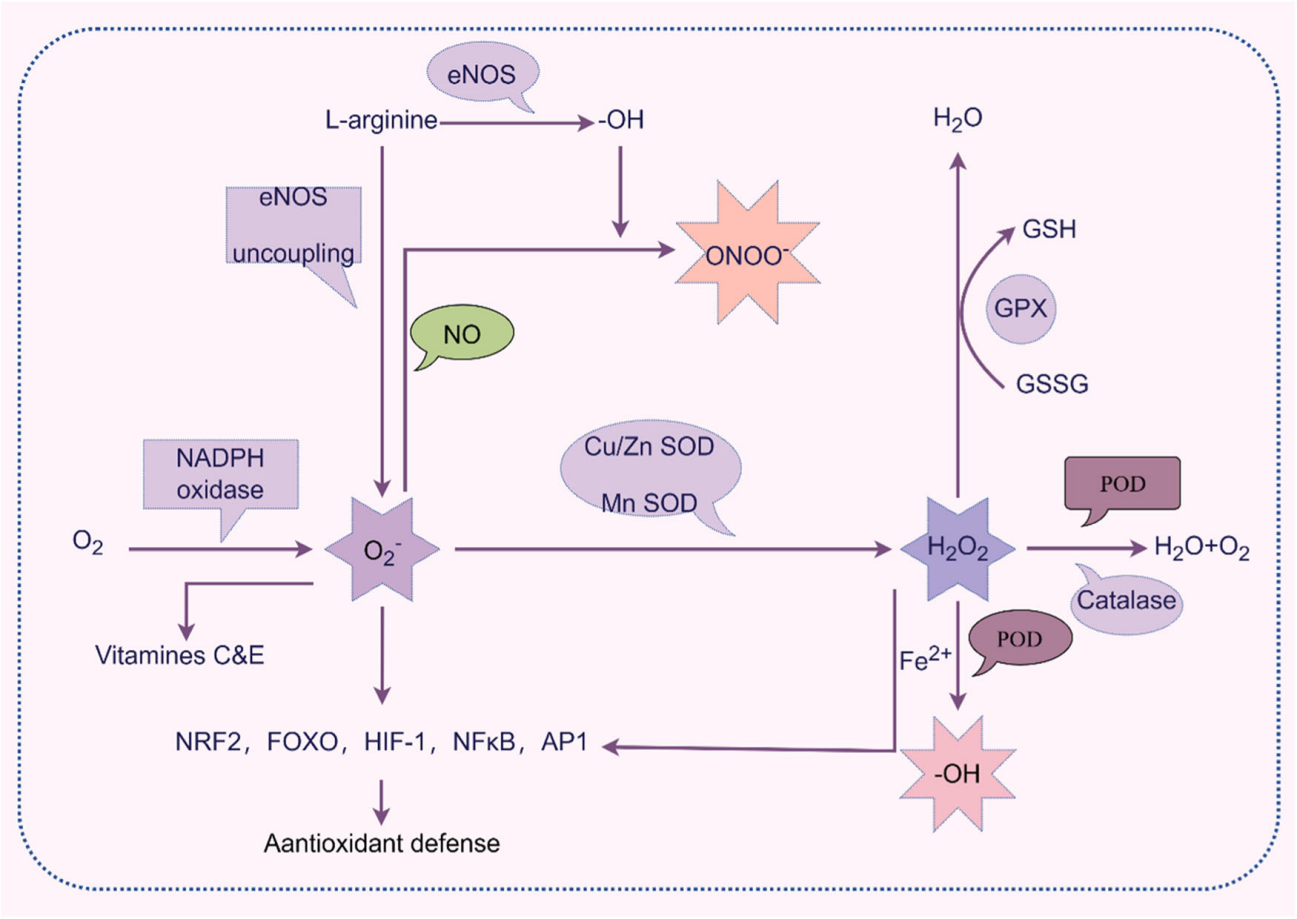
Overview of oxidative and antioxidant processes. In some conditions, under the action of Nitric Oxide Synthase (NOS) and Nicotinamide Adenine Dinucleotide Phosphate (NADPH) oxidases, superoxide anions (O_2_^−^) are formed within the body. These O_2_^−^ are rapidly converted into H_2_O_2_ by the enzyme superoxide dismutase (SOD). H_2_O_2_ is then decomposed into H_2_O, oxygen (O_2_), or hydroxyl radicals (·OH) through the action of enzymes like catalase (CAT), glutathione peroxidase (GPx), and peroxidase (POD). H_2_O_2_ participates in various signaling pathways of the oxidation and antioxidant systems. These mechanisms collectively maintain redox balance, protecting the organism from oxidative stress (OS) damage.

Neurobiological mechanisms potentially associated with TRS include dopamine super-sensitivity, hyperdopaminergic subtypes, normal dopaminergic subtypes, dysregulation of glutamate and serotonin, oxidative stress (OS), and inflammation^7^. A previous study suggested that combined deletion polymorphisms of glutathione S-transferase theta 1 and glutathione S-transferase mu 1 were associated with a 4.6-fold greater risk of TRS^8^. OS is a normal and complex physiological process in healthy individuals involving the production and neutralization of reactive oxygen species (ROS) and reactive nitrogen species, of which hydrogen peroxide (H_2_O_2_), catalase (CAT), superoxide dismutase (SOD), malondialdehyde (MDA), glutathione peroxidase (GSH-Px), nitric oxide synthase, and total antioxidant capacity (TAC) are important parameters of physiological functions^9^. As shown in Fig. 1. Many previous studies have reported that an imbalance of OS plays a vital role in the pathophysiological mechanism of schizophrenia. In these studies, abnormal levels of OS parameters, such as SOD, MDA and total oxidant status, were associated with the severity of clinical symptoms of schizophrenia^10–12^. Regarding the non-enzymatic antioxidant system, α-tocopherol (α-toc, vitamin E) levels are reportedly elevated during the acute phase of schizophrenia, while decreased in patients with chronic schizophrenia^13^. OS is thought to be associated with imbalances in specific neurotransmitter systems, which may exacerbate psychotic symptoms and reduce treatment responsiveness in patients with TRS^7,14^. TRS patients might have compromised antioxidative defenses, rendering them more susceptible to free radical damage that can aggravate their condition^15,16^. Long-term antipsychotic treatment may increase the production of free radicals or decrease the levels of antioxidants, disrupting the balance between oxidation and antioxidation^17^. Therefore, therapeutic strategies targeting OS, such as supplementing with antioxidants like vitamin E and N-acetylcysteine^18–20^, may benefit patients with schizophrenia and those with chronic-medicated schizophrenia (CMS).

Furthermore, accumulating evidence suggests that serum levels of redox biomarkers, such as CAT, GSH-Px, reduced glutathione (GSH), activator protein 1, TAC, kynurenine, and total oxidant status, could be useful as additional parameters to differentiate schizophrenia patients from healthy individuals^21^. Xie et al. found that plasma TAC levels were significantly lower in first-episode drug-naïve schizophrenic patients and associated with cognitive deficits in some domains^22^. TAC was reportedly elevated at both 8 and 26 weeks in schizophrenia patients who received n-3 polyunsaturated fatty acids as an add-on therapy^23^. Decreased TAC indicates impaired antioxidant defenses, providing support for the involvement of OS in the altered pathophysiology of schizophrenia.

H_2_O_2_ and hydroxyl radicals (·OH) are crucial ROS, with H_2_O_2_ playing a particularly significant role in reversible oxidation of key redox-sensitive cysteine residues of target proteins^24^. H_2_O_2_ is converted to ·OH by a variety of enzymes, such as CAT and peroxidase (POD), but can cause damage to lipids, proteins, and DNA, resulting in cellular dysfunction and even death^25^. Non-classical mechanisms mediated by H_2_O_2_ inhibit the release of dopamine that is otherwise modulated by neurotransmitters such as glutamate and γ-aminobutyric acid (GABA), and this modulation of DA release by glutamate and GABA depends on H_2_O_2_ generated downstream from AMPA receptors^26^, suggesting the potential involvement of H_2_O_2_ in the pathology of schizophrenia.

An imbalance in OS and even interactions with other mechanisms, such as neuroinflammation, in the pathophysiology of schizophrenia have, therefore, been the focal points of intense investigations. A systematic and comprehensive review showed that increased production of ROS stimulated by abnormal signals of the central nervous system, imbalance of redox potential, altered gene expression, neurotransmitter abnormalities, and immune dysfunction during OS were related to schizophrenia^27^. In glutamate-cysteine ligase modifier knockout mice, a vicious cycle between OS and neuroinflammation was found to have long-term effects on parvalbumin fast-spiking interneurons and integrity of perineuronal nets, which have been implicated in neural synchronization, in addition to cognitive, emotional, social, and sensory deficits^28^. Disrupted redox responses lead to the activation of matrix metalloproteinase-9 (MMP-9), triggering a complex interplay among MMP-9, receptor for advanced glycation end-products shedding, proinflammatory cytokine activation, and microglial cell activation^29^. This intricate network forms the link between OS and neuroinflammation.

MMP-9 is a member of the matrix metalloproteinase family, which comprises a group of zinc-dependent proteases that degrade various components of the extracellular matrix, leading to tissue remodeling and repair^30^. Tissue inhibitor of metalloproteinases-1 (TIMP-1) acts as an endogenous inhibitor that tightly controls the activity of MMP-9 and prevents excessive degradation of the extracellular matrix^31,32^. Furthermore, MMP-9 is regarded as a significant neuroinflammatory factor that plays a pivotal role in learning, memory, and cortical plasticity. Meanwhile, dysregulation of MMP-9 has been linked to multiple disorders, including schizophrenia, autism spectrum disorders, and epilepsy^33–35^. Several studies have reported that MMP-9, either alone or in conjunction with TIMP-1 or brain-derived neurotrophic factor, holds promise as a potential biomarker for specific conditions^36^.

Although multiple clinical and preclinical studies have demonstrated an imbalance in OS, dysfunction in oxidative defense, and abnormal neuroinflammation in patients with schizophrenia, the results are inconsistent. These discrepancies may be related to multiple factors, such as sex, age, cohort size, and the type of prescribed antipsychotic medication. In addition, the findings vary across different stages of the disorder, including patients with acute-phase schizophrenia, first-episode drug-naïve schizophrenia, and long-term medicated schizophrenia, as relatively few studies have specifically focused on TRS^37^.

As previously mentioned, OS parameters (i.e., H_2_O_2_, ·OH, POD, TAC, α-toc), MMP-9, and TIMP-1 may be involved in the underlying pathophysiology of schizophrenia. Therefore, male patients with TRS and CMS were recruited to determine (1) whether there are differences in OS parameters, MMP-9, and TIMP-1 between schizophrenia patients and healthy controls; (2) whether alterations to OS parameters, MMP-9, and TIMP-1 are related to the severity of clinical symptoms; (3) whether serum MMP-9 or TIMP-1 and plasma OS parameters levels are independently or interactively correlated with the clinical features of schizophrenia patients; and (4) whether OS parameters and MMP-9 and TIMP-1 levels are predictive of the prognosis of TRS. To the best of our knowledge, this is the first study to investigate the relationships among H_2_O_2_, ·OH, POD, TAC, α-toc, MMP-9, and TIMP-1, and the clinical symptoms of male patients with TRS.

## Results

### Comparison of demographic and general clinical data

The demographic information and clinical data of TRS and CMS patients and the healthy controls are presented in Table 1. There were no significant differences of age, education, BMI, and smoking status among the TRS, CMS and healthy control groups (all *p* > 0.05). Also, there was no significant differences in age of onset between the TRS and CMS groups (F = 1.428, *p* = 0.236). However, there were significant differences in the duration of illness, PANSS total score, PANSS subscale scores, and equivalent dose of chlorpromazine (all, *p* < 0.05).

**Table 1:** Demographics of the TRS, CMS, and healthy control groups.

	TRS (n = 31)	CMS (n = 49)	Controls (n = 53)	*F*/*Z*/*χ*^2^	*p*
Age (years)	40.65 (9.18)	40.61 (10.27)	40.02 (9.28)	0.063^a^	0.939
Education (years)	8.58 (2.53)	9.43 (3.02)	9.83 (3.06)	1.783^a^	0.172
BMI (kg/m^2^)	24.79 (3.80)	24.33 (3.60)	25.92 (3.07)	2.856^a^	0.061
Smoking	12 (38.7%)	29 (59.2%)	20 (37.7%)	5.551^b^	0.062
Age of onset (years)	25.58 (7.73)	27.88 (8.76)	–	1.428^a^	0.236
Duration of illness (years)	13 (10, 22)	10 (7, 15)	–	− 1.98^c^	0.048
PANSS total score	74.03 (7.35)	47.55 (7.96)	–	222.819^a^	<0.001
P subscores	14.03 (5.60)	9.12 (2.22)	–	30.353^a^	<0.001
N subscores	35.06 (4.64)	25.55 (4.19)	–	89.962^a^	<0.001
G subscores	24.94 (3.75)	13.33 (4.76)	–	132.125^a^	<0.001
Equivalent dose of chlorpromazine (mg/d)	675 (615, 825)	500 (430, 565)	–	− 7.51^c^	<0.001

*BMI* body mass index, *PANSS* positive and negative syndrome scale. a, one-way of analysis of variance; b, *χ*^2^ test; c, Mann–Whitney *U*-test.

### Plasma levels of OS parameters, MMP-9, and TIMP-1

The results of multivariate analysis of covariance showed that the effect of diagnosis was significant (Wilks’s lambda, F = 6.719, *p* < 0.001). ANOVA revealed significant differences in the variables H_2_O_2_ (F = 5.068, *p* = 0.008), ·OH (F = 31.856, *p* < 0.001), POD (F = 14.043, *p* < 0.001), α-toc (F = 3.711, *p* = 0.027), TAC (F = 24.098, *p* < 0.001), log MMP-9 (F = 3.219, *p* = 0.043), and log TIMP-1 (F = 0.604, *p* = 0.548) among the TRS, CMS, and healthy control groups (Table 2).

**Table 2:** Plasma levels of H_2_O_2_, ·OH, POD, and TAC of the TRS, CMS and healthy control groups.

	TRS (*n* = 31)	CMS (*n* = 49)	Controls (*n* = 53)	F	*p*
H_2_O_2_ (mmol/L)	67.06 (29.80)	62.33 (20.16)	79.20 (31.53)	5.068	0.008
·OH (U/μL)	5.20 (0.50)	5.01 (0.50)	5.82 (0.57)	31.856	<0.001
POD (U/mL)	14.32 (3.84)	11.12 (3.55)	15.51 (5.04)	14.043	<0.001
α-toc (μg/mL)	8.62 (3.65)	9.70 (3.80)	11.17 (4.99)	3.711	0.027
TAC (mmol/L)	0.70 (0.13)	0.67 (0.12)	0.82 (0.09)	24.098	<0.001
MMP-9^◄^ (ng/mL)	1.37 (0.38)	1.37 (0.32)	1.22 (0.32)	3.219	0.043
TIMP-1^◄^ (ng/mL)	1.49 (0.13)	1.51 (0.11)	1.52 (0.10)	0.604	0.548

H_2_O_2_, hydrogen peroxide; ·OH, hydroxyl radicals; POD, peroxidase; α-toc, α-tocopherol; TAC, total antioxidant capacity; MMP-9, matrix metalloproteinase-9; TIMP, tissue inhibitors of metalloproteinases. ◄, the result of natural logarithm transformations.

**Fig. 2:**
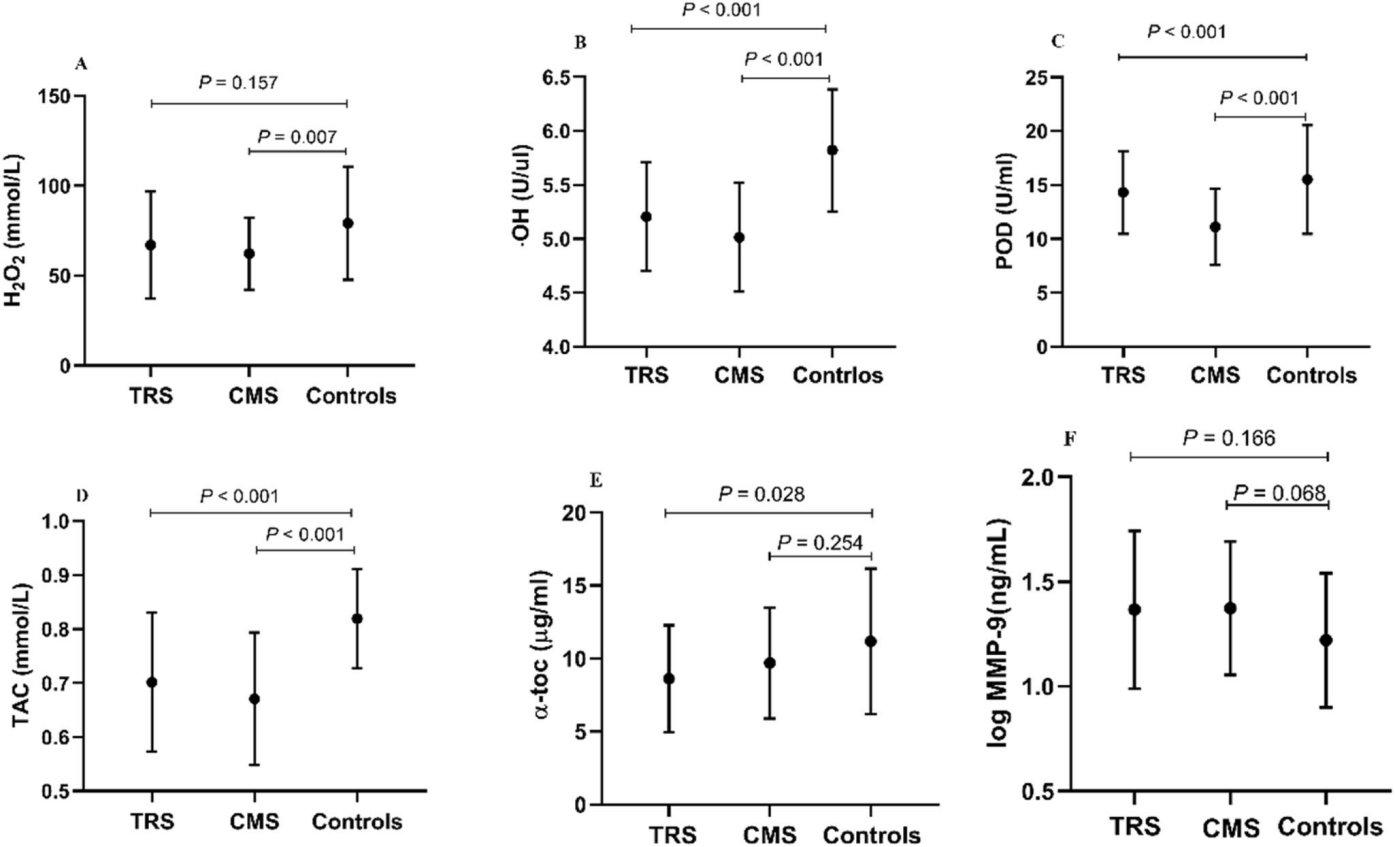
Comparisons of plasma levels of OS parameters and MMP-9 among the TRS, CMS, and healthy control groups. **A** H_2_O_2_, hydrogen peroxide; **B** ·OH, hydroxyl radicals; **C** POD, peroxidase; **D** TAC, total antioxidant capacity; **E** α-toc, α-tocopherol; **F** log MMP-9, logarithmic matrix metalloproteinase-9. TRS treatment-resistant schizophrenia, CMS chronic-medicated schizophrenia.

Post *hoc* comparisons with the Bonferroni correction method indicated that H_2_O_2_ levels were significantly lower in CMS patients than the healthy controls (*p* = 0.007), ·OH and TAC were significantly lower in TRS and CMS patients as compared to the healthy controls (*p* < 0.001), and α-toc levels were lower in TRS patients than the healthy controls (*p* = 0.028), while there were no significant differences in H_2_O_2_, ·OH, α-toc, and TAC levels between patients with TRS and CMS (all, *p* > 0.05). Additionally, POD activities were significantly decreased in CMS patients as compared to TRS patients (*p* = 0.004) and healthy controls (*p* < 0.001), while there was no significant difference between the TRS patients and healthy controls (*p* = 0.442). After Bonferroni correction, there were no significant differences in log MMP-9 and log TIMP values (all, *p* > 0.05) (Fig. 2).

Furthermore, the results of analysis of covariance revealed significant differences in plasma levels of ·OH (*F* = 27.852, *p* < 0.001), POD (F = 12.742, *p* < 0.001), α-toc (*F* = 4.141, *p* = 0.018), and TAC (*F* = 20.895, *p* < 0.001), and log MMP-9 values (*F* = 3.123, *p* = 0.047) among the TRS, CMS, and healthy control groups after correction for age, education, BMI, and smoking status. However, there were no significant differences in levels of H_2_O_2_ and log TIMP among the TRS, CMS, and healthy control groups after multiple corrections (*F* = 2.962 and 0.754, *p* = 0.055 and 0.472, respectively).

### Correlations among OS parameters, MMP-9, and TIMP-1 and clinical symptoms in TRS and CMS patients

**Fig. 3:**
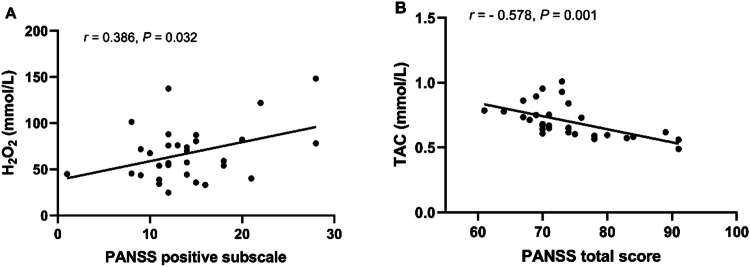
Relationships among H_2_O_2_, TAC, and clinical symptoms in TRS patients. **A** H_2_O_2_ concentration was associated with the PANSS positive symptoms score; **B** TAC was significantly negatively associated with the PANSS total score. H_2_O_2_ hydrogen peroxide, TAC total antioxidant capacity, TRS treatment-resistant schizophrenia.

For TRS patients, correlation analyses showed that the H_2_O_2_ concentration was associated with the PANSS positive symptoms score (*r* = 0.386, *p* = 0.032) (Fig. 3A) and smoking status (*r* = −0,412, *p* = 0.021), while POD activities were positively correlated with BMI (*r* = 0.412, *p* = 0.021) and TAC was significantly negatively associated with the PANSS total score (*r* = −0.578, *p* = 0.001) (Fig. 3B) and weakly positively correlated with BMI (*r* = 0.357, *p* = 0.049). Levels of α-toc were significantly correlated with age of onset (*r* = 0.525, *p* = 0.002). After controlling for confounders, multiple regression analysis revealed that the H_2_O_2_ concentration was positively correlated to the PANSS positive subscale (*B* = 0.380, *t* = 2.171, *p* = 0.039), while TAC levels were negatively significantly correlated to the PANSS total score (*B* = −0.010, *t* = −4.132, *p* < 0.001) and α-toc levels were associated with age of onset (*B* = 0.183, *t* = 2.505, *p* = 0.018). However, there were no significant associations among ·OH, POD, log MMP-9, and log TIMP-1 and PANSS total score and the PANSS subscales (all, *p* > 0.05).

**Fig. 4:**
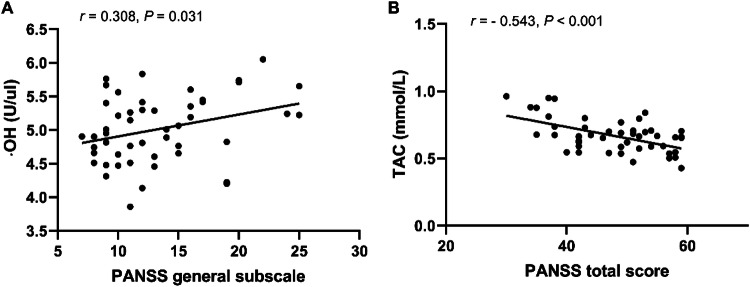
Relationship among ·OH, TAC, and clinical symptoms in CMS patients. **A** **·**OH levels were positively associated with the PANSS subscale scores; **B** TAC was negatively correlated to the PANSS total score. **·**OH hydroxyl radicals, TAC total antioxidant capacity, CMS chronic-medicated schizophrenia.

For CMS patients, ·OH levels were positively associated with the PANSS subscale scores (*r* = 0.308, *p* = 0.031) (Fig. 4A), while TAC was negatively correlated to the PANSS total score (*r* = −0.543, *p* < 0.001) (Fig. 4B). The log MMP-9 value was negatively associated with age of onset (*r* = −0.301, *p* = 0.036). There were no correlations among the H_2_O_2_, POD, and α-toc levels, log TIMP-1 values, PANSS total score, and PANSS subscale scores (all, *p* > 0.05). After controlling for confounding factors (i.e., age, years of education, BMI, smoking status, age of onset, duration of illness, and chlorpromazine equivalent dose), multiple regression analysis revealed that ·OH was associated with the PANSS subscale scores (*B* = 0.033, *t* = 2.233, *p* = 0.031), TAC, and PANSS total score (*B* = −0.008, *t* = −4.438, *p* < 0.001). The log MMP-9 value was correlated with age of onset (*B* = −0.011, t = −2.161, *p* = 0.036).

### Independent variables predictive of TRS

TRS and healthy controls were defined as dichotomous variables, modified Poisson regression analysis demonstrated that H_2_O_2_ (B = −0.008, RR = 0.992, 95%CI:0.986–0.999, *p* = 0.021), α-toc (*B* = −0.113, RR = 0.893, 95%CI: 0.834−0.956, *p* = 0.001), and ·OH (*B* = −0.328, RR = 0.265, 95%CI: 0.143−0.493, *p* < 0.001) were independent variables protective of TRS, while TAC (*B* = −2.084, RR = 0.124, 95%CI: 0.013−1.230, *p* = 0.075) and POD (B = 0.037, RR = 1.037, 95%CI: 0.964−1.116, *p* = 0.326) were not observed. With TRS and CMS being considered as dichotomous variables, modified Poisson regression analysis revealed that POD (*B* = 0.113, RR = 1.120, 95% CI: 1.031−1.217, *p* = 0.007) was a risk factor for TRS.

## Discussion

The present study showed that (1) levels of H_2_O_2_, ·OH, POD, α-toc, and TAC were decreased, and MMP-9 levels were increased in male patients with TRS and CMS as compared to healthy controls; (2) H_2_O_2_ levels in patients with TRS were positively associated with the PANSS positive psychopathology subscore, TAC was negatively correlated with the PANSS total score, ·OH activities were positively association with the PANSS general psychopathology subscore, and TAC was negatively correlated to the PANSS total score in patients with CMS; and (3) H_2_O_2_, α-toc, and ·OH may be protective factors against TRS, and POD was a risk factor. To date, this is the first study to report the relationships among H_2_O_2_ and ·OH levels, TAC, and clinical psychopathology in male patients with TRS.

In contrast to previous studies that measured antioxidant enzyme activities^12,38,39^, the current study directly assessed levels of peroxidation products, which could serve as more direct indicators of damage to the antioxidant system. In vivo, two primary sources of H_2_O_2_ are mitochondrial production and superoxides generated during oxidation of nicotinamide adenine dinucleotide phosphate (NADPH) by NADPH oxidase in the presence of SOD^40^. H_2_O_2_ can follow two pathways: (1) conversion to non-toxic substances through the actions of CAT and GSH, and (2) conversion to ·OH via POD^25,41^. When either pathway is disrupted, OS occurs, leading to various psychiatric disorders^42,43^. Mitochondrial dysfunction plays a pivotal role in the OS observed in schizophrenia, potentially serving as both a precursor and a consequence of the disorder^14^. Genetic and environmental factors induce OS within neurons, leading to mitochondrial anomalies and a compromised inflammatory response, which in turn generate further OS and neuronal damage^14,28,29^. A pathological “vicious cycle” among OS, neuroinflammation, and mitochondrial dysfunction may underlie the development of schizophrenia. Our previous studies indicated that inflammatory cytokines and OS may be involved in the pathophysiology of TRS and chronic stable schizophrenia^44^, and future studies will focus on the interplay between these factors and mitochondrial dysfunction.

Additionally, POD plays a significant role in innate immunity, apoptosis, and cell signaling, while abnormal peroxidase activities contribute to oxidative damage to cells and tissues, leading to the development of various diseases^45^. A previous study by our group found that plasma CAT activities and decreased levels of GSH-Px, SOD, and MDA were elevated in TRS and chronic patients as compared to healthy controls^44^. In the current study, H_2_O_2_ and ·OH levels were significantly reduced in TRS and CMS patients as compared to healthy controls, while POD activity was significantly decreased in CMS patients as compared to healthy controls. Moreover, levels of α-toc, a non-enzymatic antioxidant, were decreased in patients with TRS. It is noteworthy that previous study has found that the serum OS markers, H_2_O_2_ and MDA, are twice as high in obese women compared to those of normal weight and that they increase as the BMI increases^46^. Although the analyses included correction for BMI, we postulated that a higher BMI could be a contributing factor to the elevated H_2_O_2_ and ·OH levels observed in the healthy control group as well as TRS and CMS patients. Our study provides a perspective that abnormalities in oxidative and antioxidant responses may have some connection with the pathophysiology of TRS and CMS. However, as this study is cross-sectional, such associations cannot be directly interpreted as causal relationships, and these links could potentially be subject to bidirectional interactions or confounding factors. Future research should seek to verify these findings through more rigorous control of confounding factors or longitudinal studies.

Moreover, plasma levels of TAC were decreased in TRS and CMS patients, although these findings were not consistent across different types of schizophrenia. A previous study reported no significant difference in TAC levels among drug-free, medicated, and short-term-treated schizophrenia patients versus controls^12^. In contrast, serum TAC levels during admission and discharge were lower in patients with acute paranoid schizophrenia and chronic disease than those experiencing a first episode of schizophrenia^21,47^. Plasma Trolox-equivalent antioxidant capacity was comparable between treatment-responsive patients and the control group^48^. Prior research also noted decreased total antioxidant status in both drug-naïve first-episode and chronic schizophrenia patients^22,49^. Various factors can contribute to variations in OS parameters, including age, diet, smoking, sample size, assay sampling method (e.g., serum or plasma), disease stage, antipsychotic medication, illness duration, and sex^50,51^. Variations in TAC may potentially indicate compensatory effects or past OS damage within cells and may be influenced not only by various confounding factors, but also the dynamic states of antioxidant enzymes^39^. Therefore, these studies from different perspectives provide further evidence of the relationships between dysregulation of oxidative defense system and schizophrenia. In the present study, TRS was associated with changes to TAC, although the exact pathophysiological process remains unclear, thus warranting further investigations.

Furthermore, in this study, plasma MMP-9 levels were elevated in TRS patients, thereby providing an important supplementary finding to previous research^52^. The close relationship between MMP-9 and schizophrenia has been validated in various studies, including human and animal investigations of the expression and activity of MMP-9 in the central nervous system, synaptic plasticity, and gene polymorphisms^53^. In line with the findings of Yamamori et al.^54^, the results of the present study found that MMP-9 was not significantly correlated to the PANSS total score or PANSS subscale scores. As a possible explanation for these findings, MMP-9 activity may be regulated by TIMP-1, as suggested by previous research^36^. Additionally, Niitsu et al. proposed that peripheral blood levels of MMP-9 in male schizophrenia patients are associated with older age and smoking^55^. These findings indicate the need for further studies.

OS is reported to significantly impact the clinical characteristics of schizophrenia^56^. In this study, decreased H_2_O_2_ levels were positively correlated to symptoms of TRS, while decreased TAC levels were negatively associated with the total PANSS score of patients with TRS and CMS. Meanwhile, ·OH, a metabolite of H_2_O_2_, was positively associated with general symptoms of CMS. H_2_O_2_ is widely recognized as a direct indicator of OS and TAC is considered an indirect marker of OS. Imbalances between antioxidant enzymes, such as CAT, SOD, and GSH-Px, and levels of H_2_O_2_ and MDA contribute to OS, while antioxidant treatment has been shown to ameliorate the symptoms of schizophrenia^57,58^. In a previous study, doxycycline administered with antioxidant and anti-inflammatory agents reduced ketamine-induced schizophrenia-like behavior and synergistically improved the therapeutic efficacy of the antipsychotic risperidone in a murine model of schizophrenia^59^. Li et al. reported that decreased plasma TAC levels in schizophrenia patients were associated with deficits in cognitive function^60^. These results suggest that H_2_O_2_, and TAC may play important roles in the severity of clinical symptoms, indicating that changes to H_2_O_2_, and TAC levels are correlated with the pathophysiology of TRS. However, a previous study reviewed the range of chemical constituents, both direct and indirect, in cigarette smoke that may have pro-inflammatory effects and disrupt the equilibrium between antioxidants and oxidants, thereby precipitating OS^61^. The study conducted by Xiu et al. showed that smoking affects the activities of SOD, GSH-Px, and CAT, thereby influencing their predictive association with the improvement of clinical symptoms in patients with schizophrenia before and after treatment^62^. We still should take into account that smoking status may confound the relationship between H_2_O_2_ concentration and PANSS positive symptoms.

Further analysis revealed that decreased TAC levels were associated with the psychopathology of TRS, suggesting that disruption of antioxidant capacity may be associated with the occurrence of TRS. However, numerous factors that have been linked to TRS contribute to the difficulty of treatment, such as social isolation, pre- and postnatal inflammation, younger age of onset, family history, smoking, sex, types of prescribed antipsychotic medications, disease classification, clinical profiles, neuroimaging, and neurobiological factors^3,6,63^. Therefore, these findings may provide new perspectives for alternative treatment strategies for TRS.

Previous studies have indicated that α-toc supplementation can alleviate motor retardation in schizophrenia patients treated with haloperidol^23,64^, while higher or lower TAC levels reflect the degree of functioning of the oxidative defense system^65,66^. The present study provides evidence of the protective effects of TAC and α-toc against TRS, suggesting that reduced TAC and α-toc levels indicate compromised functioning of the oxidative defense system in TRS. On the other hand, POD and ·OH are influenced by H_2_O_2_ and various enzymes, although further studies are needed for confirmation. Nonetheless, these findings support the involvement of OS in the pathophysiology of TRS.

Interestingly, TAC levels were positively correlated to BMI in TRS patients. Previous studies have suggested associations between TAC and BMI in women, indicating a potential link between endogenous oxidative and antioxidant enzyme activities and lifestyle or metabolic factors^67–69^. A systematic review and meta-analysis reported a significant impact of BMI on the detection of 8-hydroxy-2-deoxyguanosine, a biomarker of oxidative DNA damage^70^. Brain imaging studies of schizophrenia and BMI have suggested that OS may contribute to decreased prefrontal myelin formation, while obesity may be associated with alterations to the frontal and temporal regions, as well as ventricular structures^71,72^. These overlapping findings provide valuable insights into the potential relationships among OS, metabolic factors, and brain structures in the pathophysiology of schizophrenia. On the other hand, existing research indicates that obesity may disrupt antioxidant enzymes^73^, with schizophrenia patients showing altered SOD and MDA levels related to weight changes post-antipsychotic treatment^74^. and a BMI-clinical symptom correlation in males, as identified by Wei et al.^75^. Although our study statistically established a relationship between TAC and BMI, and found no direct relationship between BMI and PANSS scores, it is important to consider that BMI may act as a confounding factor in the association between TAC and clinical symptoms.

There were several limitations to this study that should be addressed. First, male patients with TRS and CMS exhibited longer durations of clinical symptoms and had more compounding factors as compared to first-episode and drug-naïve patients with schizophrenia. Second, the sample size in this study was small and numerous parameters were tested. Although the Bonferroni correction method was employed to correct for multiple comparisons between groups, and several findings showed highly significant differences, the results reported for this manuscript warrant further validation with large samples to confirm these findings and mitigate the risk of potential Type II errors. Third, the causal relationships between OS parameters and PANSS scores could not be determined through cross-sectional studies, thus longitudinal studies are required. Additionally, analysis of the demographic data of only males potentially limits the generalizability of the results.

## Conclusion

In conclusion, these preliminary findings provide evidence that the levels of OS parameters (i.e., H_2_O_2_, ·OH, POD, α-toc, and TAC) and MMP-9 significantly differed among male patients with TRS and CMS. Variations in H_2_O_2_, ·OH, and TAC levels were associated with the severity of clinical symptoms. H_2_O_2_, α-toc, and ·OH were identified as protective factors against TRS. These results provide additional evidence that OS and MMP-9 may be involved in the pathophysiology of TRS and CMS.

## Subjects and methods

### Subjects and assessments

Participants were recruited from the Mental Disorder Department of the Fourth People’s Hospital of Lianyungang (Lianyungang, China). Diagnoses of schizophrenia were confirmed using the Structured Clinical Interview of the Diagnostic and Statistical Manual-IV. Patient data, including age, education, smoking status, body mass index (BMI), age of onset, and illness duration, were collected through a questionnaire. Dosages of antipsychotic medications were standardized to chlorpromazine equivalents. The severity of psychotic symptoms was assessed by two experienced psychiatrists utilizing the positive and negative syndrome scale (PANSS), with an inter-rater correlation coefficient exceeding 0.8. The study protocol was approved by the Ethics Committee of the Fourth People’s Hospital of Lianyungang and written informed consent was obtained from all participants or their guardians.

The TRS group was defined as conforming to the following criteria: (1) poor effects of two different antipsychotics medications for at least 6 months with a minimum dose of chlorpromazine at ≥600 mg/day or equivalent and (2) scores of each of the six items of the PANSS (P1, P2, P3, N1, N4, and N6) ≥ 3 and PANSS total score ≥70^3,76–78^. The CMS group was defined as having received (1) treatment with one antipsychotic medication with stable disease symptoms for >6 consecutive months with chlorpromazine at <600 mg/day or equivalent and (2) scores of each of the six items of PANSS (P1, P2, P3, N1, N4, and N6) < 3 with a PANSS total score <60^76–79^.

Patients with comorbid neurological disorders (e.g., mental retardation, dementia, epilepsy, degenerative disease, and/or traumatic brain injury) and endocrine disorders (e.g., thyroid dysfunction, diabetes mellitus, and/or alcoholic or substance dependence/abuse) were excluded from the study.

Fifty-three healthy controls, matched for age, sex, education, BMI, and smoking status, were recruited from the Lianyungang community through advertisements. Healthy controls who met the Diagnostic and Statistical Manual-IV Axis I criteria for a major disease or had a family history of mental disorder or alcohol abuse/dependence were excluded from the study. Physical examinations and laboratory tests were conducted to assess the health status of all participants.

### Blood sampling and biochemical assays

Blood samples were obtained from healthy controls and patients in the morning between 07:00 and 09:00 following overnight fasting. Blood samples were collected in anticoagulant-coated tubes and centrifuged at 3000 rpm for 15 min. The plasma was extracted from the anticoagulant tubes and stored at −80 °C until analysis. A technician blinded to the sample identity and clinical status performed duplicate assays for all blood samples. The coefficients of variation for OS parameters ranged from 3.4% to 7.2% for both intra- and inter-assay measurements.

Plasma levels of H_2_O_2_, ·OH, POD, α-toc, and TAC were measured using commercially available kits (Nanjing Jiancheng Bioengineering Institute, Nanjing, China) in accordance with the manufacturer’s instructions. Plasma levels of H_2_O_2_ (mmol/L), ·OH (U/mL), POD (U/mL), and α-toc (μg/mL) were measured using a colorimetric method, and TAC (mmol/L) was determined using an antioxidant capacity assay to assess ferric reducing antioxidant potential^22^. To obtain more explicit results for ·OH, the unit was switched from U/mL to U/μL. Serum levels of MMP-9 (ng/mL) and TIMP-1 (ng/mL) were measured with a Luminex liquid suspension chip detection assay (R&D Systems, Minneapolis, MN, USA) in accordance with the instructions provided by the manufacturer.

### Statistical analysis

Statistical analyses were conducted using IBM SPSS Statistics for Windows, version 19.0. (IBM Corporation, Armonk, NY, USA). The Kolmogorov–Smirnov test was used to assess the normal distribution of variables. Continuous variables with a normal distribution were analyzed using one-way analysis of variance and are presented as the mean ± standard deviation (SD). Non-normally distributed data were assessed with the Mann–Whitney U-test and are summarized as the median with 25th and 75th quartiles. Categorical variables were analyzed using the chi-square test. A probability (*p*) value < 0.05 was considered statistically significant. To address the non-normal distribution, logarithmic transformation was applied to serum MMP-9 and TIMP-1 levels, resulting in normally distributed data. Cohen’s d values were used to report the effect size, where 0.2 was considered a small effect size, 0.5 was a medium effect size, and 0.8 was a large effect size^80^.

To mitigate the risk of type I errors arising from potential interactions among multiple continuous dependent variables, multivariate analysis of covariance was conducted as the initial step. In this model, H_2_O_2_, ·OH, POD, α-toc, TAC, MMP-9, and TIMP-1 were dependent variables, with diagnoses (TRS, CMS, and healthy controls) as fixed factors, and age, years of education, BMI, and smoking status as covariates. Analysis of covariance was used to determine the significance of differences of each parameter among the TRS, CMS and healthy controls group. Multiple comparisons were adjusted using the Bonferroni correction method. Potential correlations of the normally and non-normally distributed data were identified using the Pearson’s correlation coefficient and Spearman’s correlation coefficient, respectively. Subsequently, stepwise multivariate regression analyses were conducted to evaluate the correlation between the OS parameters, serum levels of MMP-9 and TIMP-1, and PANSS subscale scores, while accounting for potential confounding variables. Relative risk factors for TRS were predicted using modified Poisson regression.

## Data Availability

The data supporting the results of this study are available upon request from the corresponding author.
